# Protocol for a novel approach to developing a single pan-specialty ‘meta-core outcome set’: An example from the field of surgical oncology

**DOI:** 10.12688/hrbopenres.14058.1

**Published:** 2025-01-31

**Authors:** Joel Tay, Jane Blazeby, Adam O'Neill, Yoon Loke, Aoife Lowery, Catherine Robinson, Bilal Alkhaffaf, Jamie Kirkham

**Affiliations:** 1 Centre for Biostatistics, Manchester Academic Health Science Centre, The University of Manchester, Manchester, UK; 2 NIHR Bristol Biomedical Research Centre, University Hospitals Bristol and Weston NHS Foundation Trust and University of Bristol, Bristol, England, UK; 3 Social Care and Society, School of Health Sciences, The University of Manchester, Manchester, England, UK; 4 Norwich Medical School, University of East Anglia, Norwich, England, UK; 5 Discipline of Surgery, University of Galway, Galway, Ireland; 6 Department of Oesophago-Gastric and Bariatric Surgery, Salford Royal Hospital, Northern Care Alliance NHS Foundation Trust, Salford, UK; 7 Division of Cancer Sciences, School of Medical Sciences, The University of Manchester, Manchester, England, UK

**Keywords:** Adverse events, Cancer, Core Outcome set, Consensus, Oncology, Surgery, Meta-COS

## Abstract

**Background:**

Understanding outcomes of surgery performed with curative intent for different cancer types allows comparisons to be made provided consistent outcomes are selected and measured. At present core outcome sets (COS) that represent the minimum outcomes measured and reported in any clinical trial for a given condition exists for six of the ten most prevalent cancer types but it is uncertain whether this can be used to inform the development of a meta-COS (core outcome set) for any cancer type requiring a surgical operation with curative intent. This paper describes our study protocol to develop a meta-COS for surgical oncology.

**Methods:**

Three stages of work will be conducted : (1) identification of a long list of outcomes including adverse events from previously published COS, a review of outcomes from trials registered with *ClinicalTrials.gov,* and focus groups with key stakeholders (inclusive of cancer patients having undergone surgery with curative intent for cancer or carers of such patients); (2) a two-round online Delphi survey including clinicians, patients or carers, and allied health professionals (such as dietitians, specialist nurses, physiotherapists, occupational health workers) to prioritise the outcomes; (3) an online consensus meeting using to agree on the final meta-COS.

**Discussion:**

The meta-COS for surgical oncology trials will ensure that a selection of relevant outcomes will be available for use in all research studies for any cancer type requiring a surgical intervention.

**Registration:**

This study titled “A meta-Core Outcome Set (COS) for surgical oncology” is registered on the COMET (Core Outcome Measures in Effectiveness Trials) Initiative database. ( https://www.comet-initiative.org/Studies/Details/3252).

## Introduction

Surgical oncology is a branch of medicine that treats cancer using surgery. Cancer remains the leading cause of death worldwide ( https://www.who.int/news-room/fact-sheets/detail/cancer) and surgery remains the single independent treatment modality most commonly required to treat cancer (Supplementary Figure 1 – Extended Data). In the UK, Public Health England’s official statistics also revealed that cancer diagnoses who received at least one type of treatment, surgery remains the single most common type of treatment (45%) compared to chemotherapy (28%) and radiotherapy (27%). As many as 76% of younger patients (under 50 years old) with cancer in the UK were treated with surgery ( Fraser *et al.*, 2020).

The primary and secondary outcomes used in clinical trials of surgical oncology can be heterogeneous. Consistency of these trial outcomes may be improved by the use of a core outcome set (COS), which represents the minimum set of outcomes that should be measured and reported in any clinical trial for a given condition ( Williamson *et al.*, 2012). A search of the COMET (Core Outcome Measures in Effectiveness Trials) database ( https://www.comet-initiative.org) (accessed 2 ^nd^ February 2023) indicate that six of the ten commonest ( WCRF, 2024) surgical cancers already have a COS with a further two developed for less common cancer types ( Table 1). These surgical oncology COS can often be categorised into three key domains: survival, adverse outcome and the impact on health-related quality of life ( Alkhaffaf & Kirkham, 2022). The remaining four commonest cancer types (breast, thyroid, liver and cervical) lack an available COS for use in trials and clinical settings with surgery as the main intervention type.

**Table 1.** Summary of existing COS in Surgical Oncology.

Cancer type	Rank by global incidence	No. of core outcomes	Outcomes in the COS			
			*Survival core outcomes*	*Impact of surgery outcomes*	*Adverse event/Harm * *outcomes*	*Other outcomes*
*Lung[( De Rooij *et al.*, 2022)	2	37	1. Overall survival 2. Progression-Free Survival 3. Cause of death	4. Financial impact 5. Difficulty swallowing (dysphagia) 6. Anorexia 7. Nausea 8. Gastric problems 9. Vomiting 10. Constipation 11. Diarrhoea 12. Pain 13. Fatigue 14. Shortness of breath/chest tightness 15. Coughing problems 16. Health Related Quality Of Life Outcomes (General) 17. Subjective wellbeing/health-related quality of life 18. Physical Function 19. Physical activity 20. Anxiety 21. Depression 22. Insomnia 23. Mobility 24. Cognitive functioning 25. Emotional functioning	26. Treatment discontinuation due to side effects 27. Treatment-related complications 28. Lung infection 29. Deep vein thrombosis 30. Haemoptysis	31. Medication compliance/ adherence 32. Patients’ personal beliefs and expectations about their illness 33. Social support 34. Tumour response 35. Duration of tumour Response (DoR) 36. Brain metastases 37. Localization of metastases
Colorectal( Mcnair *et al.*, 2016)	3	12	1. Long-term survival 2. Perioperative survival	3. Physical function 4. Sexual function 5. Faecal incontinence 6. Faecal urgency	7. Stoma rates and complications 8. Anastomotic leak 9. Surgical Site infection	10. Conversion to open operation (where appropriate) 11. Cancer recurrence 12. Resection margins
Prostate( Maclennan *et al.*, 2017)	4	12 universal + 4 surgery specific	1. Death from prostate cancer 2. Death from any cause 3. Perioperative deaths	4. Faecal incontinence 5. Bowel function 6. Stress incontinence 7. Urinary function 8. Sexual function 9. Overall quality of life	10. Bothersome or symptomatic urethral or anastomotic stricture 11. Thromboembolic disease	12. Positive surgical margin 13. Need for salvage therapy 14. Local disease recurrence 15. Distant disease recurrence/metastases 16. Disease progression
Stomach( Alkhaffaf *et al.*, 2021)	5	8	1. Disease-free survival 2. Disease-specific survival 3. Surgery-related death	4. Overall quality of life 5. Nutritional effects	6. Serious adverse events	7. Recurrence of cancer 8. Completeness of tumour removal
Oesophagus( Avery *et al.*, 2018)	8	10	1. Overall survival 2. In-hospital mortality	3. Severe nutritional problems 4. The ability to eat and drink 5. Problems with acid indigestion or heartburn 6. Overall quality of life	7. Respiratory complications 8. Conduit necrosis and anastomotic leak 9. The need for another operation related to their primary oesophageal cancer resection surgery	10. Inoperability
**Bladder( Reesink *et al.*, 2021)	10	11	1. Survival (cause) 2. Bladder cancer-free survival	3. Urinary symptoms 4. Fatigue 5. Quality of life 6. Pain 7. Activities of Daily living 8. Physical functioning	9. Complications after treatment (using severity grading scales Clavien-Dindo or CTCAE)	10. Health-status in palliative setting 11. Readmissions after treatment
***Pancreas( van Rijssen *et al.*, 2019)	12	8		1. Physical ability 2. Ability to work/do usual activities 3. General quality of life 4. General health 5. Abdominal complaints (pain/discomfort)		6. Relationship with partner/family 7. Fear of recurrence 8. Satisfaction with services/care organization
Oral cavity, Larynx, Nasopharynx (ENT) ( Íde Waters, 2018)	16, 20, 22 respectively	8	Disease-specific survival1. 2. Death related to treatment	3. Dysphagia 4. HR-QOL		5. Interventions for the management of treatment-related morbidity 6. Local control 7. Regional control 8. Distant metastatic control

*Only includes outcomes in the lung cancer COS, information variables are excluded here. **Radiation related complications in bladder cancer COS were excluded here. ***Pancreas COS was a Core Set of Patient-Reported Outcomes (COPRAC); Full representative pancreatic COS is currently being developed for April 2025

The development of a COS for each individual cancer type is resource intensive. Financial and time constraints become increasingly important considerations for researchers and is one of the most commonly cited barriers for standardising outcomes ( Williamson *et al.*, 2020). The recent examination ( Alkhaffaf & Kirkham, 2022) of available COS for surgical oncology demonstrated overlapping outcomes across the three aforementioned outcome domains (see also Table 1), supporting the feasibility of an efficient approach to develop a COS across cancer types involving surgical treatment (a meta-COS) as a starting point for use in all surgical oncology trials.

The overall aim of this work is to determine a surgical oncology meta-COS for any cancer type that involves a surgical operation with curative intent and, is intended to describe endpoints to be reported as a minimum in future effectiveness trials. Specific objectives are to:

1) Identify suitable outcomes that have relevance in surgical oncology for the most prevalent cancer types through a review and discussion of previously published surgical oncology COS, a review of a clinical trials registry and focus groups with key stakeholders;
2) Assess the importance of these identified outcomes among key stakeholders (surgical and oncology consultants, cancer specialist nurses, patients, carers, and researchers) using a two-round Delphi survey technique;
3) Conduct a consensus-building process to determine which outcomes (including harm outcomes) should be included in the final surgical oncology meta-COS;

## Study oversight

A Study Steering Committee (SSC) comprising three healthcare professionals (HCPs), an expert methodologist in COS development and one patient representative has been established. The three healthcare professionals consist of consultant oncological surgeons with track records as established academic researchers, two of whom had previous experience with COS development. The role of the SSC is to oversee the COS development study, provide feedback on the study protocol and list of outcomes, contribute to the dissemination of the online Delphi survey and to contribute to the final consensus meeting and dissemination of the COS.

## Methods

**Figure 1.**
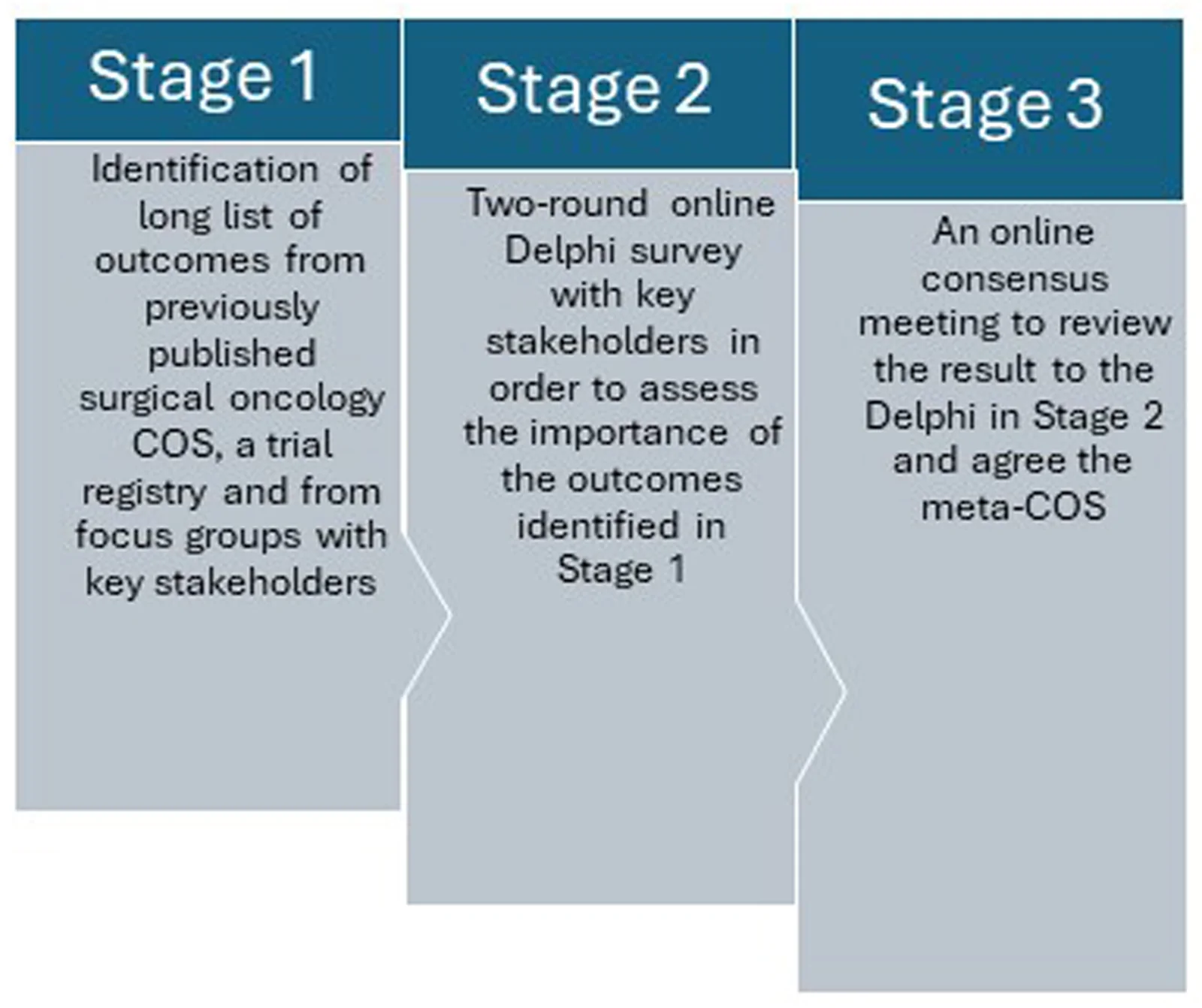
Study overview of a traditional COS development process.

The development of this COS protocol will follow the COS-STAP (Core Outcome Set-STAndardised Protocol Items) Statement ( Kirkham *et al.*, 2019), and the minimum standards for planning a COS development study ( Kirkham *et al.*, 2017). A checklist of COS-STAP items is included in the extended data for this protocol. The study is registered with the COMET Initiative ( https://www.comet-initiative.org/Studies/Details/3252). The COS process will involve three steps in Figure 1. Each of these steps is described below:

### Stage 1: Identification of outcomes

The list of outcomes from the ten most common surgical cancers reported globally will be identified (lung, breast, colorectal, prostate, gastric, liver, thyroid, cervical, bladder, oesophageal ( WCRF, 2024)) for use in an online Delphi survey. These outcomes will be generated from the following sources:

1) Outcomes that were identified in existing published surgery oncology COS which were identified from a previous COMET database search ( https://www.comet-initiative.org/Studies). From Table 1, we have identified eight previously published COS within scope which included two cancer types outside the top 10 (pancreatic and oropharyngeal (ENT) cancers), which the SSC decided to also include.
2)  For cancer types within the top ten commonest ones where there is no existing COS (breast, thyroid, liver and cervical), outcomes will be identified from a review of open and recruiting trials registered on a trials registry ( https://clinicaltrials.gov/). The search was carried out on 20 ^th^ March 2024 and will capture all records on the database. The search strategy is described in Table 2.
3) Three separate focus groups will be undertaken (one group for each type of stakeholders – patients/carers, consultant clinicians, and allied healthcare professionals) to capture any additional outcomes not already identified. Language of the outcomes and their definitions will also be checked in these group work. For each focus group meeting, the aim will be to capture participants representing as many of the different cancer types as possible.

**Table 2.** Search strategy for each cancer type on ClinicalTrials.gov.

	BREAST	THYROID	LIVER	CERVICAL
CONDITION	Breast cancer	Thyroid cancer	Hepatocellular carcinoma/Liver cancer	Cervix cancer
OTHER TERMS, IF ANY		Thyroid gland, thyroid carcinoma, thyroid neoplasm		
INTERVENTION	Surgery	Surgery	Surgery	Surgery
FILTERS	Phase 3 and 4 trials only	*Interventional trials only	Phase 3 and 4 trials only	Phase 3 and 4 trials only
	Interventional trials only		Interventional trials only	Interventional trials only
**EXCLUSION CRITERIA**	Non-surgical trials, Oopherectomy-only trials	Non-surgical trials, Metastatic thyroid cancers	Non-surgical trials,	Non-surgical trials, Different cervical (head & neck) cancer trials.,

*No additional results returned for phase 3 and 4 filter for thyroid cancer(s) on the trial registry used.

***Review of the final outcome list***

The resulting list of outcomes from Stage 1 will undergo a series of independent iterative reviews and discussion between the lead investigator and the SSC. The aim of these reviews will be to remove all duplicate outcomes and to retain only outcomes that would be deemed to be relevant for inclusion across all cancer types. As an example, ‘residual shoulder abduction deficit’ following breast cancer surgery would not be relevant for all cancer types, thereby would not be suitable for consideration in a surgical oncology meta-COS. Conversely, disease-free survival following cervical cancer surgery would be relevant across all cancer types, and thus would be included as a candidate outcome for the meta-COS. If there is uncertainty in the decision-making process, then the outcome will be retained for consideration in Stage 2. All decisions made will be documented with rationale for why that decision was made.

The final list of outcomes from all sources will be reviewed by the SSC to group similar outcomes together and categorise each according to a 38-item taxonomy ( Dodd *et al.*, 2018). Outcome descriptors will be ensured by patient and public representatives to be clear and using appropriate plain language. Particular attention will be paid to the adverse events core domain in order to capture important individual harms ( Tay *et al.*, 2024). The SSC will also discuss the level of granularity of the outcome domains and will consider options for merging and dropping outcomes to ensure it was practically feasible for participants to score all outcomes in the Delphi survey phase in Stage 2 ( Kottner *et al.*, 2024).

### Stage 2: Outcome prioritisation

***Stakeholder recruitment***

Stakeholders representing health care professionals (HCPs), researchers and patients will be invited to participate in the consensus building process. Healthcare professionals will include consultant surgeons, consultant oncologists, cancer specialist nurses, dietitians, and physiotherapists. They will be divided into 3 broad stakeholder groups – consultants (surgeons or oncologists), allied health professionals (nurses, therapists and dietitians) and patients (or carers of). We will look to include at least two participants from each of these stakeholder group for each of the ten most commonest cancer types.

Participants will be invited through professional networks and charities via email and social media

For example, HCPs will be identified through national societies, regional cancer networks, and regional trial units. Examples of these include, but not exhaustive of:

- Greater Manchester Cancer Alliance
- North West Surgical Trials Unit
- Association of Surgeons of Great Britain
- British Association of Surgical Oncology
- National Breast Cancer Research Institute Ireland
- National Surgical Research Support Centre (RCSI)
- Cancer Research UK

We do not intend to recruit a separate stakeholder group of non-clinical academic researchers or triallists. Instead, HCPs with experience in trials, cancer research, or experience with COS development will be preferred. The SSC will assist in screening interested HCPs based on their research outputs and any prior COS development involvement.

Patient and carer participants will be identified through charity and cancer support networks both regionally and nationally. Examples of these include, but not exhaustive of:

- Independent Cancer Patients’ Voice (ICPV)
- The Patients Association (PA)
- Greater Manchester Cancer Alliance
- National Breast Cancer Research Institute Ireland
- Irish Cancer Society
- Patient and Public Involvement (PPI) Ignite Ireland

For patients or carers of patients, only those who have completed their treatment of the cancer diagnosis will be invited to participate. We anticipate the perceived priority of outcomes whilst still undergoing treatment will differ to those having completed. The stage of the cancer will not be specified in the recruitment due to the breadth of cancer types being targeted. Instead patients who have had cancer surgery with the intention to cure will be recruited.

***Online delphi survey***

A list of outcomes identified from the sources described in Stage 1 will be prioritised in a two-round online Delphi survey. The outcomes will be presented in terms of the core areas identified by the taxonomy: survival, physiological or clinical, life impact, resource use or adverse events (AE), and subsequently ordered into the relevant domains underpinning the core areas (for example the domain “global quality of life” falls under the core area of ‘life impact’; and ‘surgical site infections’ under ‘adverse events’.

Due to the importance of reporting AEs ( Cornelius & Phillips, 2022; Junqueira *et al.*, 2023; Zorzela *et al.*, 2016), AEs will be presented as a domain separate to the other outcomes for consideration. They will not be mandated for inclusion into the final COS but participants will be encouraged to consider them by their own merits.

Participants will be asked to rate the importance of each outcome on a 9-point Likert scale (1–3 limited importance, 4–5 important but not critical and, 7–9 critical) following the GRADE (Grading of Recommendations, Assessment, Development and Evaluations) ( Guyatt *et al.*, 2011) guidelines. An option of ‘unsure’ will be included where participants feel they are unable to score an outcomes importance. Participants will also be invited to voluntarily comment on each outcome should they wish to do so. At the end of the first round, participants will have the option of adding in additional outcomes that they think are important but have not been included in the original list. Any additional outcomes will be reviewed by the SCC to consider whether these outcomes are relevant and not duplicated, and if agreed, these outcomes will be included in round two of the Delphi survey. All outcomes scored in round one will be carried forward to round two.

In the second round of the Delphi survey, participants will be presented with their individual ratings from round one and the distribution of scores for each outcome according to a) each stakeholder group and b) overall across all stakeholder groups. Participants will have the opportunity to re-review outcomes and re-score the outcomes based on this information presented using the same 1–9 scale should they wish too. Newly added outcomes will be presented in round two and participants will be asked to score these new outcomes without reflection. Retention of participants between the two rounds will be monitored and several reminders will be sent to encourage continued engagement and completion of both rounds of the survey. Attrition bias between rounds will be assessed by looking at the average distribution of scores between rounds across all outcomes for each stakeholder group. Final responses from the second round will be analysed using descriptive statistics and summarised according to predefined consensus criteria ( Table 3). The Delphi survey will be administered and managed using REDCap ( Harris *et al.*, 2009; Harris *et al.*, 2019).

**Table 3.** Consensus criteria.

Consensus Classification	Description	Definition
Consensus in	Consensus that the outcome should be included in the final meta-core outcome set	80% or more participants scoring as 7–9 (critical) in each stakeholder groups
Consensus out	Consensus that the outcome should *not* be included in the final meta-core outcome set	50% or fewer participants scoring 7–9 (critical) in each stakeholder group
No consensus	Uncertainty about the importance of the outcome	All other responses

### Stage 3: Consensus meeting

The recruitment for the consensus meeting will be aimed at individual participants who have completed both rounds of the Delphi survey. The estimated number for recruitment into this meeting would be no more than 15 with an equal breakdown of five consultant clinicians (surgeons or oncologists), five allied health professionals and five patients or carers.

Less importance will be placed on the cancer type each stakeholder represents at this stage, as the scope for this meta-COS is for all cancer involving surgery. The recruitment of this will be a direct email invitation following the completion of the Delphi survey.

The consensus meeting will be conducted interactively via Zoom under the guidance of an experienced independent facilitator. The meeting will be organised around the results to the final round of the Delphi survey. Outcomes will be prioritised for discussion if they met the consensus criteria by at least one stakeholder group and/or outcomes were scored as critically important by at least 50% of participants in each stakeholder group. Participants will be invited to briefly discuss the importance of each outcome if they wish before an anonymous vote on whether to include the outcome in the final meta-COS. For the outcome to be preliminary included in the final meta-core set, 80% or more of the participants needed to vote the outcome as critical.

***Final meta-COS for surgical oncology***

At the end of the consensus meeting, the consensus meeting panel will review the proposed outcomes to be included in the meta-COS following the discussions and voting. A final reflective discussion will be undertaken to ensure the outcomes included are pragmatic and feasible to measure for the COS’ intended purpose of use. If a final meta-COS is not agreed on at the end of the consensus meeting, subsequent online meetings will be considered in order to ratify the final meta-COS.

## Discussion

The aim of this work is to standardise the collection of core outcomes in surgical oncology. It does not replace existing COS in the field, but forms an overarching minimum set that underpins these available COS and provides a starting point for trials involving cancer types lacking a COS. This novel approach will minimise the need for researchers developing a COS for individual cancer types to traditionally perform a comprehensive literature review for a list of cancer outcomes as shown in Figure 1. This expedited focussed approach will save significant amount of resources and time.

Adverse event (AE) outcomes are important to be collected and reported. This is already advocated by regulating bodies (Health Research Authority), World Health Organisation (WHO) ( WHO, 2023), and the Cochrane Collaboration ( Peryer *et al.*, 2023). Recommendations for COS development exist ( Kirkham *et al.*, 2017), albeit with little guidance to ensure important harms are included in COS when appropriate ( Tay *et al.*, 2024). The need to improve the way these cancer COS are developed have already been recognised ( Gargon *et al.*, 2019).

Whilst the inclusion of AE outcomes in trials and COS is variable ( Tay *et al.*, 2024), information on AEs is crucial so that safety is not misrepresented in any particular cancer intervention. Our approach in presenting a separate AE domain to be considered in the meta-COS development process will ensure that stakeholders have time to consider the important of each AE outcome, even if they do not result in inclusion in the final meta-COS.

One limitation of this study is by selecting only the commonest 10 cancer types for surgical oncology as representation for all cancer types we could potentially miss certain outcomes. However this is estimated to be low risk given the focus of most of the surgical oncology COS has been on survival and life impact. Another limitation is that the majority of recruitment networks engaged are in the North West of England due to the location of the research team. Efforts will be made to recruit using national charities and in Ireland according to requirements of the grant body for this project.

The uptake of core outcome sets vary widely across health conditions ( Hughes *et al.*, 2021). One of the common barriers of uptake often relates to the lack of consensus on the instruments that should be used to assess each core outcome. A formal assessment of appropriate instruments for measuring each core outcome was beyond the scope of this study. Future work will be needed to reach a consensus on these outcome measures.

## Dissemination

The results of this study will be disseminated via presentations to charity and network groups, presentations at scientific meetings and publications in open access journals.

## Ethics and consent

This consensus study has been granted for approval by the Proportionate University Research Ethics Committee (UREC) on the 29 ^th^ October 2024. Reference: 2024-21348-37826. The focus group in the earlier stage was granted a separate full ethics approval by the UREC on the 7 ^th^ March 2024. Reference: 2024-18269-33406.

Written consent will be obtained from all participants in this consensus study after they have read the participant information sheet. The consent statements participants need to agree to are embedded into the beginning of the survey and mandatory for proceeding. All data will be anonymised in the reporting of this study. Transcripts of the consensus meeting will also be anonymised. Consent can be withdrawn at any time however any entered data will not be possible to be removed.

## Data Availability

No data are associated with this article. Open Science Framework: Protocol for developing a single pan-specialty 'meta-core outcome set': an example in surgical oncology, https://doi.org/10.17605/OSF.IO/CSZ9X ( Tay *et al.*, 2025). This project contains the following extended data: 1. COS-STAP checklist for this protocol 2. Consent form for Delphi survey 3. Example PDF of Delphi survey Round 1 4. Participation Information Sheet for Healthcare Professionals 5. Participation Information Sheet for patients and carers 6. Supplementary Figure 1 - Taken from Cancer Research UK digital app. Data are available under the terms of the Creative Commons Zero "No rights reserved" data waiver (CC0 1.0 Public domain dedication).
