# Supplementary material for: Protocol for a novel approach to developing a single pan-specialty ‘meta-core outcome set’: An example from the field of surgical oncology
Source: HRB Open Res. 2025 Jan 31;8:19. [Version 1] doi: 10.12688/hrbopenres.14058.1 (PMC13583348; doi:10.12688/hrbopenres.14058.1)
Supplement: Peer review history

# Reviewer report: Reviewer response for version 1

**DOI:** 10.21956/hrbopenres.15437.r56786

**Published:** 2026-09-16

**Authors:** Munir Tarazi

**Affiliations:** 1 Imperial College London, London, UK

This manuscript describes a protocol for the development of a surgical oncology meta-Core Outcome Set (meta-COS), an overarching minimum set of outcomes intended for use across all cancer types involving surgery with curative intent. The topic is timely, clinically relevant, and methodologically important. The heterogeneity of outcomes used in surgical oncology trials is a well-recognised and a significant problem, and the development of a meta-COS represents a pragmatic and resource-efficient solution. The three-stage methodology: outcome identification, Delphi prioritisation, and consensus meeting, is broadly appropriate and follows established COS development guidance.

Is the study design appropriate for the research question?

Yes

Is the rationale for, and objectives of, the study clearly described?

Yes

Are sufficient details of the methods provided to allow replication by others?

Yes

Are the datasets clearly presented in a useable and accessible format?

Not applicable

Reviewer Expertise:

Oesophagogastric surgery, postoperative outcomes

I confirm that I have read this submission and believe that I have an appropriate level of expertise to confirm that it is of an acceptable scientific standard.

# Reviewer report: Reviewer response for version 1

**DOI:** 10.21956/hrbopenres.15437.r47411

**Published:** 2025-06-02

**Authors:** Wasay Nizam

**Affiliations:** 1 Howard University College of Medicine, Washington, USA

This is an appropriate undertaking. Surgical oncology is an evolving field and adding this will streamline future research endeavors. Interpretation of existing studies is limited by the heterogeneity of outcomes described. Harmonizing these will suitably improve future studies. I look forward to the results of this meeting and wish the authors well.

Is the study design appropriate for the research question?

Yes

Is the rationale for, and objectives of, the study clearly described?

Yes

Are sufficient details of the methods provided to allow replication by others?

Yes

Are the datasets clearly presented in a useable and accessible format?

Not applicable

Reviewer Expertise:

Surgical oncology

I confirm that I have read this submission and believe that I have an appropriate level of expertise to confirm that it is of an acceptable scientific standard.
